# Oilseed crop sunflower (*Helianthus annuus*) as a source of food: Nutritional and health benefits

**DOI:** 10.1002/fsn3.1783

**Published:** 2020-07-31

**Authors:** Bartholomew Saanu Adeleke, Olubukola Oluranti Babalola

**Affiliations:** ^1^ Food Security and Safety Niche Area Faculty of Natural and Agricultural Sciences North‐West University Mmabatho South Africa

**Keywords:** food security, human nutrition, oilseeds, sunfoil, sustainable agriculture

## Abstract

The use of biofertilizers in developing environmentally friendly agriculture as an alternative to chemical‐based fertilizers in enhancing food production is promising in sustainable agriculture for the improvement in the yield of some commercial crops such as sunflowers and other oilseed crops in terms of quality and quantity. Sunflower is an important oilseed crop native to South America and currently cultivated throughout the world. Generally, the sunflower is considered important based on its nutritional and medicinal value. Due to its beneficial health effects, sunflower has been recognized as functional foods or nutraceutical, although not yet fully harnessed. Sunflower contains mineral elements and phytochemicals such as dietary fiber, manganese, vitamins, tocopherols, phytosterols, triterpene glycosides, α‐tocopherol, glutathione reductase, flavonoids, phenolic acids, carotenoids, peptides, chlorogenic acid, caffeic acid, alkaloids, tannins, and saponins; and these compounds contribute to their functional and nutraceutical development. The extract from sunflower is known to be a potential source of antimicrobial, anti‐inflammatory, antitumor, and antioxidants agents that protect human cells against harmful reactive oxygen molecules and pathogenic microorganisms. Also, the pharmacological survey on sunflower had revealed its curative power to different kinds of diseases. The health benefits of sunflower include blood pressure and diabetic control, skin protection, and lowering cholesterol and other functions. This review is written with appropriate referencing to previously published work and provides updated information regarding the new method of organic farming for sunflower production, nutritional and health benefits, and its by‐products as human diet and livestock feed. Also, the constraints of sunflower production are elucidated.

## Graphical abstract

Agricultural food production in conventional farming using chemical fertilizers and pesticides has pose threats to soil ecology. Sunflower and its products serve as source of nutritional food for man and livestock. Consumption of sunflower oil and seeds with high antioxidant content is beneficial in scavenging the free radicals that might likely cause disease conditions in human.

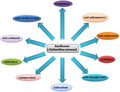

## 1 INTRODUCTION

The continuous rising in the human population and high demand for foods has resulted in hunger, disease outbreak, and even starvation to death; therefore, there is a need to intensify more on agricultural practices for maximum food production for the human populace (Pandey, 2018). Food production for human nutrition is essential for healthy living (El‐Hamidi & Zaher, 2018). Over time, many farmers have engaged in conventional agricultural practice using chemical fertilizers for better crop yields and productivity and these in a systemic way adversely affect crop yield, physical and chemical properties of soil, water as a result of surface runoff, and microbial ecological imbalance (Elemike, Uzoh, Onwudiwe, & Babalola, 2019). The persistent use of chemical fertilizers through plants has channeled into a plant–food chain system, causing disease symptoms in humans (Sharma & Singhvi, 2017). How do we avert the menaces of some of these challenges facing agriculture? It is imperative to critically devise biotechnologically modern approaches needed, suitable in the agricultural system in improving crop yields, and productivity devoid of threats to the environment (Grieve et al., 2019).

The exploitation of microbial resources in formulating bioinoculants (biofertilizer and biopesticides) has contributed to safe food product delivery in the agronomic system sustainably (Mahanty et al., 2017). The soil amended with microbial‐based formulated fertilizer (biofertilizer) promotes plant health and crop yield with intents of proffering long‐lasting solutions to the problems associated with the continuous use of chemical fertilizers in enriching soil fertility for crop productivity (Adeniji & Babalola, 2019). The use of biofertilizers as applied to some crops such as maize, legumes, tubers, and oilseeds crop has been reported (Babalola & Glick, 2012; Igiehon & Babalola, 2017; Mukherjee, Tripathi, Mukherjee, Mallick, & Banerjee, 2019; Win, Barone, Secundo, & Fu, 2018). Thus, growing of plant‐based high‐quality foods such tuber crops, oilseed crops (sunflower, rapeseed, safflower, and soybeans), fruits, and vegetables has been domesticated as a source of diet for humans and raw materials for industry (Al Surmi, El Dengawy, & Khalifa, 2016; Laguna et al., 2018).

Sunflower (*Helianthus annuus*) is an oilseed crop native to North America. It is cultivated throughout the world, and most of its products have been commercialized as culinary or livestock feed (Yegorov, Turpurova, Sharabaeva, & Bondar, 2019). The adaptation of sunflowers to different climatic and soil conditions has enhanced its cultivation as an oilseed plant throughout the world (Forleo, Palmieri, Suardi, Coaloa, & Pari, 2018). The growth of sunflower requires fertile soil, moderate rainfall, viable seeds, etc. Among the three leading oilseed crops, that is, soybean, rapeseed, and sunflower in the world today, sunflower has been recognized as a major source of high‐quality edible oil importantly used for culinary purposes (Pal, Patra, Sahoo, Bakhara, & Panda, 2015).

Sunflower is one of the important oilseed crops grown throughout the world as a source of premium oil and dietary fiber that significantly contributes to human health (Khan, Choudhary, Pandey, Khan, & Thomas, 2015). In some countries like India and South Africa, growing of sunflowers might be more competitive to other crops like maize, soybean, and sorghum (Vijayakumar et al., 2016). Due to the continuous increase in the human population, the demand for edible sunflower seeds, oil, and by‐products has also increased, and to meet the demand, there is a need to intensify efforts to expand sunflower output (Taher, Javani, Beyaz, & Yildiz, 2017). Today, the international oilseed market is dominated mainly by sunflowers and other oilseed crops such as soybean, rapeseed, peanut, cottonseed, etc.

The nutritional components of sunflowers are numerous. Examples are sunflower meal, cake, etc. The sunflower meal or cake representing a unique by‐product obtained from the extracted sunflower‐processed seeds accounts for 36% mass composition, protein content ranging between 45% and 50% (Malik & Saini, 2018; Malik, Sharma, & Saini, 2017). Sunflower meal is composed of essential amino acids, vitamin B and minerals, and high antioxidant property, which is fascinating as a nutritional food for humans and composite meals for livestock (Wanjari & Waghmare, 2015). Although, the use of sunflower meals in the human diet is limited due to the presence of anti‐nutrients (saponins, protease inhibitor, and arginase inhibitor), insoluble fiber and presence of a trace of residue solvents in the meal after extraction (Grasso, Omoarukhe, Wen, Papoutsis, & Methven, 2019). Conversely, adequate processing of sunflower seeds has caused a reduction in its anti‐nutritional contents, thus making it importantly safe for human consumption (Adesina, 2018).

As a promising protein source, sunflower seeds in food preparation can be made as a substitute to soybean, where its production is limited (de Morais Oliveira et al., 2016). Sunflowers have been employed in the preparation of various delicacy as seed, in the processed or extracted form, or form of composite products (Adesina, 2018). Sunflower seeds can be processed into different forms, such as flour, roasted, baked, or boiled as composite functional foods (Grasso et al., 2019). Sunflower remains a source of nutritional food for humans. Studies have revealed that sunflower seeds are rich nutrients and certain different phytochemicals such as antioxidants, flavonols, phenolic acids, procyanidins, phytosterols, amino acids, dietary fiber, potassium, arginine monounsaturated, and polyunsaturated fatty acids which contribute to the improvement of human health (Alagawany, Farag, Abd El‐Hack, & Dhama, 2015; Guo, Ge, & Jom, 2017; Islam, Hossain, Majumder, & Tipu, 2016). According to literature, the antioxidant activity of phenolic compounds in sunflower seed has been reported (Menzel, González‐Martínez, Chiralt, & Vilaplana, 2019; Ye, Liang, Li, & Zhao, 2015). Ye et al. (2015) had reported caffeic acid hexose I, caffeic acid hexose II, p‐Coumaric acid hexose, chlorogenic acid, isoquercitrin, 3,4‐Di‐O‐caffeoylquinic acid, 1,5‐Di‐O‐caffeoylquinic acid, 3,5‐Di‐O‐caffeoylquinic acid, and 4,5‐Di‐O‐caffeoylquinic acid as principal phenolic contents in sunflower florets. The presence of some essential amino acids such as aspartic acid, glutamic acid, serine, histidine, glycine, threonine, arginine, alanine, tyrosine, cysteine, valine, methionine, phenylalanine, isoleucine, leucine, lysine, and proline in sunflower products has also been reported (Karangwa et al., 2015). Guo et al. (2017) had reported various phytochemical biological compounds possessing multifunctional activities from sunflower.

In recent time, the research into harnessing sunflower by‐products has increased, and lack of detailed documentation on its nutritional and health benefits has contributed to their underutilization (Karefyllakis, van der Goot, & Nikiforidis, 2019). The oil obtained from the processed sunflower seeds has been used in many homes for cooking and as a raw material in the food industry in the production of margarine, butter, bread, and snacks (Kottapalli et al., 2020). The price of oilseed crops might be competitive in the market as compared to other seed oils. Hence, the biological ingredients in sunflowers can add more value for consumer choice based on its importance on human health (Franco, Iseppi, & Taverna, 2018). The processed sunflower seeds are low in carbohydrates but contain high proteins, dietary fiber, and fatty acids, as well as sources of antioxidants, vitamins, and minerals (Shahbaz et al., 2018). The nutritional composition of sunflower seeds and oil has dictated their functional properties, also effective in preventing or controlling human diseases such as diabetes, cancers, hypertension, hypercholesterolemia, and coronary heart disease (Katsarou et al., 2015). Today, we can say that the use of sunflower oil for culinary purposes that form a major part of the human diet in many homes may perhaps be due to the knowledge about the nutritional composition of sunflower oil, an increase in the human population and concerted approach toward healthy living.

Sunflower has been used as a composite ingredient in bread and butter production (Martins, Pinho, & Ferreira, 2017). Aside production of edible oil, sunflower has been used as raw materials in the production of cosmetics, paints, lubricants, biodiesel, and drugs (Rocha‐Filho, Alves, Maruno, Ferrari, & Topan, 2016). The nutritional and economic importance of sunflower oil is very paramount. The antioxidants contents can be adjudged based on its as important functional foods with many health benefits. Hence, it may be considered as a therapeutic dose in the management, prevention, and control of frittering diseases associated with reactive oxygen systems (Cao & Mezzenga, 2019). Sunflower seeds oil is rich in oleic acid and can be rated as potential vegetable oils in the human diet (Alberio et al., 2016). Due to the high protein and nutritional composition of sunflower seeds, it may contribute to enhancing human dietary protein intake (Dadalt, Velayudhan, Neto, Slominski, & Nyachoti, 2016), although sunflower seeds might not primarily meet for edible protein, rather its oil content for various industrial commercialization.

Owing to the presence of certain natural compounds in plants, this has been the major research focus of some scientists with deeper knowledge into nutritional and bioactive compounds of sunflowers, and from the previous studies, the high antioxidant activity and other nutritional value in sunflower have been reported (Alagawany et al., 2015; Karangwa et al., 2015; Malik & Saini, 2018). Sunflower possesses antitumor, anti‐inflammatory, antioxidant, skin‐protective, anticancer, antimalarial, hypocholesterolemic, antihypertensive, analgesic, and antimicrobial activity and silent effects on muscles, nerves, and blood vessel (Zoumpoulakis, Sinanoglou, Siapi, Heropoulos, & Proestos, 2017). Based on the pharmacological properties of sunflower seeds and oil, it can be recommended as a potential treatment dose in the prevention of diseases affecting humans. Thus, various parts of sunflowers have been found efficient in traditional medicines as a healing therapy in the treatment of dysentery, cough, skin rashes, diarrhea, sores, etc. (Mohiuddin, 2019). From studies so far, little information has been documented on the pharmacological and nutritional aspects of sunflower. Therefore, this review aimed at elucidating the production, nutritional, and health benefits of sunflower oils and seeds as essential ingredients in the human diet and livestock feeds.

## 2 SUNFLOWER

Sunflower is a short season plant classified into family Asteraceae and genus *Helianthus* with more than 70 species known worldwide. The name “sunflower” is derived from its size and the image of the plant, which resembles the sun. Also, the rotation around the sun is the origin of the name. Sunflower is characterized by large‐circular yellow inflorescence flower head (bearing achenes developing into mature seeds) facing directly the rays from the sun, long taproot, hairy stems, broad, coarsely toothed, and rough leaves. It originated from native temperate climates (temperature ranging between 20 and 25°C) of North America and was later introduced into Europe by Spanish explorers in the sixteenth century. The flora part of sunflowers reveals its esthetic and ornamental values with varied sizes and colors, changing from cream to yellow among different cultivars (Vilvert, Lana, Zander, & Sieber, 2018).

Globally, the sunflower is ranked the fourth most important oilseed crop after soybeans, rapeseed, and safflower, as the most profitable and economic oilseed crop. It is primarily produced for the development of high‐oil varieties by plant scientists under favorable conditions or maximum yield and productivity that requires fertile soil, adequate rainfall, and suitable environmental conditions. Nutrients limiting, unsuitable environmental conditions such as climatic, edaphic, and managerial factors can cause a reduction in the yield of sunflower products such as seeds, oil content, and other products. The application of organic manure plus synthetic fertilizer could influence the yield and quality of sunflower. The supply of essential micronutrients like potassium increases crop productivity as well as crop tolerance to drought and environmental stress (Enebe & Babalola, 2018).

Presently, the sunflower is one of the most leading oilseed crops in the Southern part of Africa. It is commonly grown in the summer season with adequate rainfall. The yields of sunflower production varied annually, depending on the climatic conditions and market prices. Growing and survival of sunflower plants under different soil conditions make them compete favorably as an alternative to other cereal grains such as maize, sorghum, or cowpea. In comparison to other cereal grains, sunflowers grow maximally under high temperatures and drought. Moisture conservation due to long, deep taproot system enables the plant to recover rapidly from moisture loss, and for survival under stress conditions (Hussain et al., 2018).

Sunflowers contribute about 87% of vegetable oil production, making it preferred over other oilseed crops. It forms an economical and promising agricultural crop with many benefits in enhancing valuable market products, provide a source of income and poverty alleviation. However, the lack of viable seeds available to farmers and harsh weather conditions have caused limitations in harnessing its full potential along food value chains. The yield can be explored maximally as an alternative to the existing oil crops such as palm oil, palm kernel oil, soybean, rapeseed, and peanut if properly harnessed (Bassegio et al., 2016).

Sunflowers have emerged as an economical oil crop that can be incorporated into local cropping systems, enhance soil health, and increased biodiversity in a crop rotation system. The plants have strong adaptive mechanisms in growing in complex environments; it does not require high levels of fertility as do maize, wheat, and other crops to enhance their yields. Aside from edible oil production, sunflowers are food plants that can be eaten raw, roasted, cooked, dried, and ground. The roasted seeds can serve as a substitute for coffee. From a horticultural point of view, sunflowers are regarded as multipurpose ornamentals plants that can be planted for recreational purposes, wildlife game, an abode for birds and rodents; and source of food and income for the man.

Large quantities and varieties of sunflowers are produced worldwide. Two types of sunflowers are known, namely: (a) oilseed type and (b) confectionary type. In terms of color, the oil‐producing seed is black with a thin hull adhering to the kernel. The oilseeds contain high oil content compared to the nonoilseed type used for confectionary purposes. The oil from sunflower oilseeds is considered safe and suitable for human consumption with low cholesterol. Edible oilseeds required in the human diet could comprise of sunflower oil, canola oil, and soybean oil. The oils from the oilseeds can be used as a composite ingredient in food preparation. The confectionary or nonoilseed is larger than the oil‐type sunflower with seed colors being black, white, and black with white stripes. The nonoilseeds are important and are normally used in rather small doses (Eryilmaz & Yesilyurt, 2016).

Oilseeds are mainly derived from oil‐producing plants. The four major oil crops in the world include rapeseed‐mustard, soybean, sunflower, and oil palm, their production per metric million tones is represented (Table 1). The demand for edible and nonedible oils in underdeveloped, developed, and developing countries keep expanding, with the motive of income generation from the domestic output and as a boost to the world economy. The most beneficial aspect of oilseeds from sunflower is built around its ability to produce substantive and quality oil. Current researches are ongoing in applying bioinformatics and biotechnological approaches in growing sunflower for maximum yield in terms of seeds and oils. The nutraceutical tendencies of sunflower oil as a functional food could significantly help in developing products with long‐term effects in reducing the risk associated with diseases on the consumers (Aremu, Omotayo, & Babalola, 2016).

**TABLE 1:** World production of oilseed crops

Oilseed	Production (million metric tons)					
	2013/14	2014/15	2015/16	2016/17	2017/18	2018/19
Sunflower	38.56	39.18	40.36	48.01	47.39	51.46
Soybean	308.40	320.72	345.97	345.97	360.08	360.08
Cotton seeds	46.70	44.36	35.76	39.08	44.99	43.45
Rape seed	71.00	70.42	68.74	69.43	74.00	70.91
Oil palm	56.38	59.30	61.75	58.88	70.46	73.49

### 2.1 Sunflower oil (Sunfoil)

Sunflower oil is generally classified as nonvolatile oil obtained during the processing of sunflower seeds for oil production. It is often time used as principal ingredients in food preparation such as frying and formulation of cosmetics (emollient). As of 2017, the world production of sunflower oil was approximately 16 million tonnes with the largest production from Ukraine and Russia. Correspondingly, it was reported in 2014 that the world production of sunflower oil attained 15.8 million tonnes with the production rate per countries as follows: Ukraine is producing 4.4 million tonnes, followed by Russia, 4.1 million tonnes; Argentina, 0.9 million tonnes; Bulgaria, 0.8 million tonnes, while Turkey recorded least 0.7 million tonnes (Lai et al., 2017).

The major composition of sunflower oil is linoleic acid (polyunsaturated fat) and oleic acid (mono‐saturated fat). The variation in the fatty acid content of sunflower oil might be due to the plant species and the processing treatment employed during its production. The oil is attributed to light amber coloration and fascinating flavor. The oil is rich vitamins and mineral elements. Production and commercialization of sunflower oil can better be achieved using modern technology to meet consumer demand for high‐quality oil.

Sunflower oil contains 5% palmitic acid, 6% stearic acid, 30% oleic acid (monounsaturated omega‐9), and linoleic acid (polyunsaturated omega‐6) with 59% (Avni, Anupriya, Rai, Maan, & Naryansamy, 2016). Also, other sunflower oils produced via plant breeding and industrial processing include high‐linoleic with 69% linoleic acid, high‐oleic with 82% oleic acid, mid‐oleic with 65% oleic acid, and high‐stearic with high‐oleic containing 18% stearic acid and 72% oleic acid respectively (Gupta, 2014).

Many oil seeds growing in tropical Africa are yet to be explored due to a lack of knowledge about their nutritional and economic values. Currently, edible oil from sunflowers and other oilseeds crops is globally dominating the world market, most especially in South Africa (Antonopoulou, Vayenas, & Lyberatos, 2016). To meet demand, an increase in production is required to ensure the maximum supply of high‐grade edible oil to the increasing population. In South Africa, the high demand for sunflower oil from the domestic market due to its rate of consumption accounts for approximately 8% of total oil production of the National oil requirements when compared to other oilseeds crops. Sunflower oil is not only produced for human consumption alone but also as raw materials for industries. Therefore, there is an increasing demand for sunflower oil in meeting up with the dietary requirements around the world. In the year 2015–2016, the production and consumption of sunflower‐based products (seeds) in the world amounted to approximately 15 million metric tons (Wang, Zhu, Li, Wei, & Sun, 2019).

Sunfoil as an agricultural product can be obtained naturally from most oilseed plants for various uses. The oil value chain can be enhanced for the well‐being of an individual and the world economy. The enzymatic reactions and high temperatures that occur during storage can cause the oil to go rancid. Oil serves as the main energy food with 9.3 calorie/g in the living cells. The breakdown of fats and oils in the living system yields fatty acids and glycerol. Oilseeds from plants have been used extensively in cooking due to the less cholesterol and unsaturated nature, which helps in preventing likely human heart diseases.

Edible oils have been extracted from the sunflower oilseeds, canola oilseeds, soybean oilseeds, cotton seeds, groundnut/peanut seed, linseed/flaxseed oil, sesame seed, rapeseed, jatropha seeds, jojoba seeds, and castor oilseeds, etc which are categorized as essential and fixed. Essential oil is volatile, usually obtained from nonseed parts of the plants used in the production of perfumes, flavors, deodorants, antiseptics, and pharmaceuticals, while the fixed oils are derived from the seeds of oil plants, edible with high nutritional value (Blasi et al., 2018).

Sunflower oil (SFO) is the nonvolatile oil extracted from healthy sunflower seeds. It is used as a cooking oil with unique physical and chemical properties. After production, it remains liquid at room temperature and can stand on the shelf for more than one year in the market. It has potential applications in the cosmetic industry because of its water retention and noninflammable (Oliveira et al., 2019). SFO like other oil crops contains carotenoids, tocopherols, tocotrienols, and sterols, with light‐yellowish coloration containing high polyunsaturated fatty acids (linoleic acid—65%) and mono‐saturated oleic acid (70%), this accounts for 48%–74% of the total fatty acids of sunflower oil with low levels of saturated fatty acids—palmitic and stearic acids (15%) unlike other seed oils such as soybean and rapeseed which contain less amount of linoleic acid (El‐Hamidi & Zaher, 2018). The consumption of sunfoil helps in maintaining low‐density lipoprotein and cholesterol levels in the body, which is advantageous in the treatment of disease conditions like acne, arthritis, and hair damage. Sunflower oil is widely consumed and is the preferred oil in most of Europe, Mexico, and several South American countries due to easy accessibility and health benefits. The oil extract from sunflower serves as excellent phenolic antioxidants, which account for about 1%–4% of the total mass chlorogenic acid. It also contains phytosterols, which help in the alteration of cholesterol synthesis, thereby reducing the cholesterol level in the serum through cholesterol excretion (Zoumpoulakis et al., 2017).

### 2.2 Sunflower meal

Sunflower meal is synonymous with sunflower oil meal, sunflower seed meal, dehulled sunflower meal, un‐dehulled sunflower meal, decorticated sunflower meal, etc. It is one of the by‐products obtained from sunflower seeds processing. In terms of nutritional balance, it is ranked the 4th most invaluable oil meal after cottonseed meal, rapeseed meal, and soybean meal. In agro‐markets, varieties of inexpensive by‐products from cereals ranging from low‐quality straw‐like meal to high‐quality flours are available. Sunflower meals can be processed from whole, dehulled, or decorticated seeds through the mechanical or solvent extraction method, and these determine the quality of the resulting sunflower products.

Sunflower meal is the largest by‐product obtained after the oil extraction process from sunflower seeds in an industry. It forms an excellent source of protein for human consumption and as a supplementary diet in ruminant and nonruminant feeds, which enhance the animal growth and milk production as well as relative biomass generation. It serves as a rich source of proteins the sulfur‐containing amino acids (cysteine and methionine) and other essential amino acids like leucine, valine, isoleucine, tryptophan, alanine, phenylalanine, and valine; mineral and vitamins such as phosphorus, thiamine, nicotinic acid, pantothenic acid, riboflavin, and biotin help in muscle growth and development (Laguna et al., 2018). It is sometimes considered to be nutritionally inferior to rapeseed meal, cottonseed meal, and soybean meal owing to its high fiber and lower protein and metabolizable energy levels. Also, oil cake is one of the by‐products of sunflower seeds after extraction. It contains 50% protein, which is essential as a supplement in animal feeds. It contains high fiber content with a similar value equivalent to 72% soybean oil cake. Sunflower cake is used in South Africa and other countries like Tanzania as the main component of livestock feeds. In Tanzania, due to their small livestock small industry, they usually export sunflower cake to India and Kenya for financial value in exchange (Mmongoyo et al., 2017).

Sunflower meal is available worldwide. In 2019, the world production estimate of sunflower meal was 21.8.5 million tonnes, with Ukraine producing approximately 7 million tonnes and Russian Federation 5.1 million tonnes. The European Union (EU‐27) is ranked 3rd major producer 4.8 million tonnes of sunflower meal (Rahoveanu, Rahoveanu, & Ion, 2018). After the oil extraction from the sunflower seeds, the resulting by‐product (sunflower meal) is usually air‐dried to remove the trace of moisture content before storage. Additionally, molding into shapes, ground, or form pellet makes it easier to handle and preserve by adding a suitable carbohydrates or fats stabilizer under high pressure in a pelletizer or extruder machine.

Solvent extraction of sunflower meal was first commercially available, not until two decades now that the use of mechanical pressure becomes more popular with the advent of organic farming and on‐farm oil production. Solvent extraction by extrusion reduced the fat content of the processed seeds. Notably, the dehulling of sunflower seeds reduced the fiber content, correspondingly with high protein content. The decorticated sunflower meals have high protein and low fiber, while partially decorticated meals and nondecorticated meals said to be low in protein with high fiber, though the distinction between the meals is unknown. Sunflower meal is sold or graded based on its protein content like other protein feeds sources such as soybean meal, fish meal, and safflower meal. In some countries of the world like the USA, proper labeling the carry manufacturer's information is ascribed on the product and made available to the customer about the compositional quality of the sunflower meal. The color of the sunflower meal usually changes from gray to black, depending on the dehulling and extraction processes. Processed sunflower meal with the fewer hull is usually lighter in color and form excellent ingredients in the feed of livestock majorly rabbits, poultry, and pigs (Alagawany et al., 2015).

### 2.3 Sunflower seeds

**FIGURE 1:**
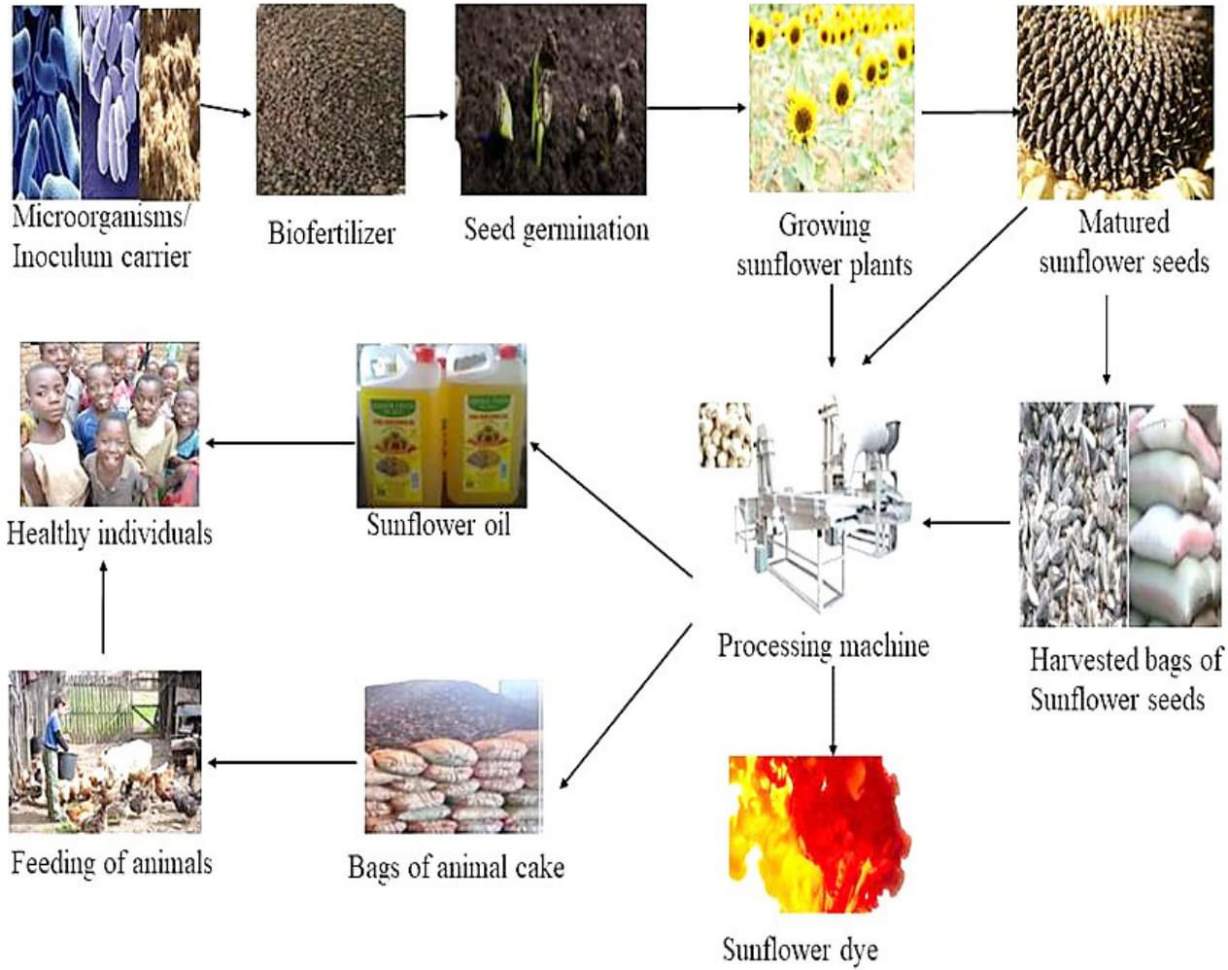
Sunflower processing and derivable products

Sunflower seeds are one of the world's leading oil‐producing seeds. The plants are hardy and drought‐resistant, well suited to colder or arid areas where many other nonoilseed crops cannot survive. After maturation, sunflower seeds are usually exposed at the apical part of the plant. The seeds are made up of the outer part called epicarp, middle layer mesocarp, and an inner layer called the endocarp. The seeds are confined within an achene consisting of a shell composed of lignin and cellulolytic materials covering the kernel, which accounts for 80% of its total weight. Raw sunflower seeds usually contain about 25% oil, but through plant breeding, it has increased to 40%. The seed oil can be extracted by cold extraction and hot‐pressing. Cold press oil is usually used in salad dressing, cooking, and margarine production, while the hot‐press involving thermal extraction is mainly used for derivable products such as paints, soaps, detergents, and pest adjuvant in industries (Figure 1). At maturation on the open field, birds feed on sunflower seeds because of proteins and fats they contained. It has been documented that the size of the farm determines the level of damage that could be done by a given population of birds on the sunflower seeds yield at a given time.

Sunflower seeds contain high amounts of vitamins like vitamin E, B, folate, and niacin and minerals like calcium, copper, iron, magnesium, manganese, selenium, phosphorous, potassium, sodium, and zinc. By and large, the therapeutic potential of sunflower seeds has been proven medically curative for colds and coughs, as a substitute for quinine, exhibiting anti‐malaria efficacy and as a diuretic and expectorant (Islam et al., 2016).

During harvesting, care must be taken in uprooting the stalk of the plants as topsoil containing nutrients needed for plant growth might be attached to the stalk. Harvesting and storage of sunflower seeds can be facilitated by dry weather. This makes farmers prefer planting sunflowers toward the end of the year (around October to early November), which is generally followed by a long spell of sunshine. Storage of sunflower seeds is essential after harvesting from the farm. The seeds can be processed by removing the kernel before being stored like grains. Proper storage and low relative humidity of the storage environment must be ensured to prevent postharvest contamination with aflatoxigenic molds (Omotayo, Omotayo, Mwanza, & Babalola, 2019).

Sunflower seeds production is the major concern of most sunflower farmers in the developing countries, due to its multi‐nutritional components such amino acids, proteins, unsaturated fats, fiber, vitamins E, and mineral elements (selenium, copper, zinc, folate, and iron), which has made it a domesticated food source in many homes. Dehulled and roasted sunflower seeds are rich in methionine and cysteine which could serve as alternative nutritious meals for man and in feeding livestock. Sunflower seeds contained up to 20% protein, reserved proteins that supply essential nutrients (sulfur and nitrogen) for seedling development after germination (Muhammad Anjum, Nadeem, Issa Khan, & Hussain, 2012).

The amino acid content glutamic acid (26.91), aspartic acid (10.50), arginine (9.75), phenylalanine, tyrosine, leucine (8.57), methionine (6.18), and cysteine (3.47) of sunflower seeds have been reported (Ivanova, Chalova, Koleva, & Pishtiyski, 2013). The blending of sunflower seed flour with other cereal crops adds value and improves food protein contents. The oil contents extracted from sunflower seeds are more in linoleic acid (55%–70%) than the oleic acid (20%–25%). Sunflower seeds displayed high antioxidants as anti‐cholesterol with low‐density lipoprotein tendency. Besides, it contained polyunsaturated fatty acids (31.0%) which is higher when compared to other oilseeds like safflower seed (28.2%), sesame (25.5%), fax (22.4%), cottonseed (18.1%), peanut (13.1%), and soy (3.5%) (Saunders, Davis, & Garg, 2013).

### 2.4 Chemical and nutrients component of sunflower seeds

In recent times, the awareness by nutritionists, dietetics, food chemists on balance dietary intake to prevent health‐related problems such as obesity, cardiovascular, and neurological diseases have led to high demand for vegetable oil such as sunfoil, as a major ingredient in the human diet with characteristic flavors and textures. The physical and chemical composition of edible oils depends primarily on the biological origin, and they contain dietary fiber, minerals, vitamins (vitamin E), and antimicrobial and antioxidant agents. Over time, the consumption of foods with high saturated fatty acids content can affect certain organs in the human body, causing obesity, cancer, and diabetes. The functional properties of foods can be improved by biological and chemical processes. Lipids from plant origin are considered healthier and more beneficial than fats from animal sources due to their high polyunsaturated fatty acids that are essential in organ functioning, improve health, and reduce the incidence of life‐threatening ailments such as cardiovascular diseases. Therefore, oils from oilseeds crops (soybean, sunflower, olive, and rapeseed) are found as major sources of edible oils in the global world market for human consumption with high Ω‐3 and Ω‐6 fatty acid contents as well as other biological components (Van‐Nieuwenhove et al., 2019).

The fatty acids profiles of sunfoil can be classified as low‐oleic acid, medium‐oleic acid, and high‐oleic acid. Most of the oilseed crops contain polyphenols as endogenous antioxidants that prevent lipid oxidation. Natural antioxidants from the bioactive components of foods and the predominant phenolic compound in sunflower seeds are chlorogenic acid (Li et al., 2018). The chemical constituents of sunflower seeds contained flavonoids which include heliannone, quercetin, kaempferol, luteolin, apigenin; phenolic acids such as caffeic acid, chlorogenic acid, caffeoylquinic acid, gallic acid, protocatechuic, coumaric, ferulic acid, and sinapic acids and fatty acids (lauric, palmitic, oleic, linoleic, stearic, linolenic, and heneicosanoic). The most widely occurring substitution patterns for flavones are 5,7,4‐trioxygenation (apigenin type) and 5,7,3,4‐tetraoxygenation (luteolin type) while flavonols include 3,5,7,4‐tetraoxygenation (kaempferol type) and 3,5,7,3,4‐pentaoxygenation (quercetion type) (Guo et al., 2017). Some plants contain natural antioxidants which help in scavenging free radicals against toxic molecules, reducing the risk of chronic diseases, and cellular damage. Natural antioxidants from plants could be categorized as enzymes (catalase, glutathione dehydrogenase, and guaiacol peroxidase), peptides (reduced glutathione), carotenoids, and phenolic compounds (tocopherols, flavonoids, and phenolic acids).

### 2.5 Sunflower extracts

The sunflower extracts are rich in carbohydrate (sugars), phytosterols, toxic alkaloids, flavonoids, saponins, glycoside, steroids, amino acids, proteins, fruits acids, and mineral salts. The application of sunflower extracts is found invaluable in the food and pharmaceutical industries. The active biological extracts from stem, leaf, roots, and seeds of sunflower can be obtained using polar and nonpolar solvents. The activity of the extract yield depends on the nature of the plant matrix and the extracting solvents (chloroform, ethanol, *N*‐hexane, methanol, etc.). Polar solvents such as methanol, butanol, and ethanol in their real state or an aqueous mixture are commonly used for the extraction of phenolic components from plant material (Mansouri, Mirzabe, & Ráufi, 2017). A single solvent can be used, but the maximum extract yield of the active plant components might be reduced; therefore, the combination of plant extraction solvents can be used to obtain the maximum yield of the plant extract. Oil extracted from sunflower seeds is considered healthy and can minimize the risk of cardiovascular disease. Sunflower has been used in the manufacturing of paints, adhesives, fabric softener, lubricants, coatings, varnishes, plastics, soaps and detergents, formulation of bio‐pesticide, carrier for agrochemicals, and surfactants in industries. It is used in salad dressing and also as an alternative source of fuel in automobile (diesel) engines (Table 2).

**TABLE 2:** Economic importance of sunflower

Area	Importance	References
Food	Blend of high linoleic and oleic sunflower oil with selected cold‐pressed oils Tocopherols and phytosterols containing antioxidants. Sunflower products as a source of proteins	de Figueiredo, Fernández, and Nolasco (2019) Farahmandfar et al. (2018)
Animal feed	Sunflower products fed to ruminants, birds, and fishes. Protein hydrolysis using proteases. Nutritional value of sunflower on livestock. Livestock feed formulation in South Africa	de Morais Oliveira et al. (2016)
Energy	Source of biofuel (biodiesel). Bioenergy generation. Premium and quality oil production	Azad, Rasul, Khan, Sharma, and Islam (2015)
Health	Health benefits of sunflower seeds. Health benefits of sunflower oil. Low cholesterol levels improving health. Antioxidant potentials of sunflower seeds.	Zoumpoulakis et al. (2017) Cheng et al. (2019)
Sustainability	Economic sustainability of sunflower production. Sunflower cultivation using organic fertilizer. Sustainable sunflower processing. Use of biofertilizer for food safety and security. For production on functional and improved seed varieties.	Amiri, Asgharipour, Campbell, and Armin (2019)
Industry	Production of edible oil with low cholesterol. Production of dyes, paints, surfactants. Formulation of nutritionally cheap livestock feeds	Lai et al. (2017) Martins et al. (2017)

The oil from sunflower seeds constitutes ingredients used in cooking, manufacture of soaps, detergents, and varnish and lighting oil. From time immemorial, sunflower crop has been exploited and used for many purposes such as ornamental plants (religious ceremony), food (roasted kernels and flour), medicine (anti‐inflammatory and diuretic), baking; thicken soups and stews, as well as making a coffee‐like beverage. The dried hulls, pollens, and petals are used in making dyes and face paint. In ethnomedicine, it is found effective as an antidote to snake bites and in the treatment of several diseases, which include heart diseases and other related ailments such as whooping cough.

It has been reported that sunflower seeds contained high total phenolic compounds and active components tocopherols, which have been used as a stabilizer of fat‐containing foods and also aid important biological functions associated with oil bodies. Therefore, natural antioxidants from plant seeds have received great attention as a source of bioactive substances with many health benefits, acting as anti‐inflammatory, anticancer, and antimicrobial agents (Menzel et al., 2019).

## 3 BENEFITS OF SUNFLOWER

The exploration and responsive use of extracts prepared from medicinal plants are on the increase in phytomedicine. The nutraceutical functional oil, for example sunflower oil, contains unsaturated fatty acids, vitamin E, antioxidants tocopherols and tocotrienols, phytosterols, phenolics, carotenoids, and chlorophylls. The nutraceutical features of common oilseed crops have been reported (Arruda, Pereira, de Morais, Eberlin, & Pastore, 2018). Better still, the current awareness about the continuous use of synthetic pharmaceuticals in phytomedicines and extraction natural functional oil from plants as an alternative could help in proffering curable solutions to various diseases affecting humans (Maqsood, Adiamo, Ahmad, & Mudgil, 2020). Also, the use of bioactive dietary extracts from medicinal plants in disease prevention and management has been generally accepted due to their enormous health benefits.

Sunflower seeds, sunfoil, and oilseed‐extracted meals are attributed to high antioxidant properties. The shell of sunflower seeds majorly reserves phenolic compounds antioxidant. The seeds of sunflower antioxidants contain 2%–4% phenolic compounds. Sunflower kernel also accounts for 43%–73% phenolic compounds. The phenolic compound in sunflower seeds has been identified using HPLC analysis (Karamać, Kosińska, Estrella, Hernández, & Duenas, 2012). The derivable products from sunflower seeds containing invaluable antioxidants have been applied in food technology. More importantly, the beta and alpha‐carotene present in sunflower oil are similar to the pigments characterizable coloration in most plants, which include ripe tomato, banana, cashew, carrots, and other foods. The human immune system against foreign materials is enhanced by the natural antioxidants present in foods. Carotenoids and tocopherol are antioxidants found in sunflower oil that neutralize free radicals, scavenge them and prevent oxidative damage to cells or tissues, thus exhibiting antitumor, anti‐inflammatory, and cardio‐protective responses. Free radicals get into the human body system through smoking, stress, ingestion of contaminated food and water, and other environmental factors that degenerate into disease conditions such as cardiovascular disease and cancer. It is very important to feed on a diet rich in antioxidants to prevent severe damage that might be done to our body. The natural antioxidants in foods help in maintaining the pH of the body system against free radicals as protection to cellular aging and human diseases (Xu et al., 2017).

The common natural source of antioxidants such as tocopherols, flavonoids, carotenoids, phenolic acids, lignins, and tannins could be used as a replacement to synthetic additives with a vital role in the prevention of diseases. The addition of organic food additive prevents food from going rancid due to oxidation of biomolecule (fat) in foods. Food rancidity as a result of lipid oxidation reduces food quality on increases shelf life. Ingestion of food rich in antioxidants can confer health benefits to living organisms by impeding the formation or development of free radicals that are injurious to the cells. The presence of free radicals may affect the easy pathway of biologically active molecules as implicated in the onset of the disease manifestation. Studies have established that quick degeneration of food products due to redox processes can lower the immune system and easily gateway to risky infectious diseases such as diabetes, arthritis, rheumatoid, cancer, cataracts, respiratory, atherosclerosis, schizophrenia, and Alzheimer's at any age (Karamać et al., 2012).

Consumption of food and food products with high antioxidants boosts the body's immune system in fighting against foreign antigens that might cause damage to the host cell. Sunflower products are not only beneficial to humans, but the by‐product also serves as a primary source of protein in ruminants and nonruminant feeds. The multifaceted nutritional benefits of sunflower seeds do not only limited to that, but it is also useful in pharmaceutical industries. Due to the high oleic and linoleic acids in sunflower oil, its consumption has reduced the chances of high cholesterol, total cholesterol, and coronary artery diseases. The presence of phytosterols in sunflower seeds has remarkably efficient in lowering cholesterol levels, increase body immunity, and reducing the risk of colon cancer (Smith, Patterson, Walker, & Verghese, 2016). As a result, the anti‐inflammatory action of antioxidants present in sunflower seeds has made them promising in medicine in the treatment of chronic inflammatory disease conditions such as osteoarthritis, rheumatoid arthritis, and bronchial asthma.

The sunflower oil containing essential vitamin E is beneficial in lowering atherosclerosis, artery disease, and stroke. Magnesium is also an important element required for the proper functioning of body nerve and muscle. Sunflower seeds usually high in magnesium functionally reduce hypertension, migraine, muscle cramps, and bronchial asthma problems (Houston, 2014). Another important element selenium can easily be incorporated into the active site of many antioxidant enzymes like glutathione peroxidase. Fundamentally, sunflower seeds are rich in zinc, a mineral element that boosts the human immune system. Due to the numerous nutritional benefits, its efficiency in pharmaceutical has made them applicable as a cheap treatment in phytomedicine as well as a source of mineral elements required for cell functioning. In short, the various nutrients present in natural health products and functional entities like sunflower oil as nutraceutical could be beneficial in reducing the potential risk of human diseases (Islam et al., 2016).

## 4 NUTRITIONAL ATTRIBUTES AND HEALTH IMPORTANCE OF SUNFLOWER

The health benefits of sunflower seeds/oils are credited to its major nutritional constituents which include high monounsaturated and polyunsaturated fats, proteins, tocopherols, phytosterols, copper, zinc, folate, iron and vitamin B possessing antimicrobial, antidiabetic, anti‐inflammatory, antihypertensive, and antioxidants (Nandha, Singh, Garg, & Rani, 2014) (Table 3). The principal fatty acids component of sunflower oil includes oleic, stearic, linoleic, and palmitic acid. Also, sunflower oil contains carotenoids, waxes, lecithin, and tocopherols (Kozłowska & Gruczyńska, 2018). The demand for sunflower‐based food has become increasingly important, and due to its nutritional and health value, it is majorly consumed as oil food for cooking or processed seeds in several forms such as salad, snacks, butter, bread, and margarine as food (Khan et al., 2015). It can as well be roasted, salted, boiled, or fermented.

**TABLE 3:** Biological effects and constituents of sunflower seeds

Biological effect	Biological constituents
Antioxidant	Enzymes (catalase, glutathione reductase, guaiacol peroxidase, glutathione dehydrogenase), phenolic compounds (flavonoids, phenolic acids and tocopherols), carotenoids, l‐ascorbic acid, peptides
Anti‐inflammatory	Helianthosides, triterpene glycosides, α‐tocopherol
Antidiabetic	Quinic acid, glycosides, chlorogenic acid, caffeic acid, phytosterols
Antimicrobial	Alkaloids, glycosides, tannins, saponins, phenolic compounds
Antihypertensive	11S globulin peptides

Regardless of its forms, the consumption of sunflower‐based food provides consumers with appropriate nutritional and health benefits (Rauf et al., 2017). The nutritional and medicinal aspects of sunflowers have not been given much attention like other oilseed crops, although human beings depend on sunflower owing to its nutritional contents. Consequently, sunflowers can be formulated as functional food or nutraceuticals with associated health and nutritional benefits (Kumar et al., 2019). Sunflower seeds are considered to contain a low level of sugar and glycemic content in most oilseed crops as well as an excellent source of unsaturated fats (cholesterol‐free), vitamins, dietary fiber, oil, proteins and mineral elements like copper, phosphorus, manganese, amino acids, zinc, iron, folate, and vitamins (Poulsen & Blaabjerg, 2017). The beneficial health effects of some oilseed crops such as corn oil, sunflower oil, and canola and the need to include the plant oil containing high oleic and linoleic acid have been reported (Ghani, Kulkarni, Song, Shannon, & Lee, 2016; Loganes, Ballali, & Minto, 2016).

The nutritional and health benefits of sunflower and its derivable products like sunflower meal, oil, processed seeds, snacks, and extracts are known to form part of human diet and livestock feed, yet their utilization has not been fully explored. Sunflower‐based products can be used as composite food in the production of varieties of the human diet with complex nutritional indices that improve human health (Grasso et al., 2019).

From literature, it has been reported that consumption of processed sunflower as a source of a meal is therapeutically important in reducing the risk of chronic organs‐associated diseases in humans (Sarwar, Sarwar, Sarwar, Qadri, & Moghal, 2013) and this necessitates more intensification in its agricultural production and research as promising oilseed crops with multifunctional attributes. Therefore, the daily consumption of sunflower oil can be recommended to boost human immunity for healthy living.

The unique nutritional and bioactive components of sunflower have played major roles in diverse areas relating to human health, as the nutritional profile of sunflower oil is known to lower lipid content and exert anti‐inflammatory effects against some chronic diseases (Khan et al., 2015). The consumption of either whole sunflower seeds or other products such as sunflower oil, snacks, and the meal has proven to be beneficial to health, majorly in response to the lipid profiles of sunflower oil containing high unsaturated fatty acids than the saturated fatty acids (Pal et al., 2015). Based on nutritional content, the sunflower fat content contained 10.3% saturated, 19.5% monounsaturated, and 65.7% polyunsaturated (Škorić, Jocić, Sakač, & Lečić, 2008). Several studies have revealed that the fatty acid content in sunflower oil containing high oleic acid positively exhibits much influence on cardiovascular risk factors such as glucose metabolism, lipid profile, and blood pressure (Vijayakumar et al., 2016). The beneficial effects of sunflower oil rich in oleic acid have been reported (Khan et al., 2015), and similar effects have also been reported for olive oil, peanut oil, pent seeds, and its derivable products (Akhtar, Khalid, Ahmed, Shahzad, & Suleria, 2014).

**FIGURE 2:**
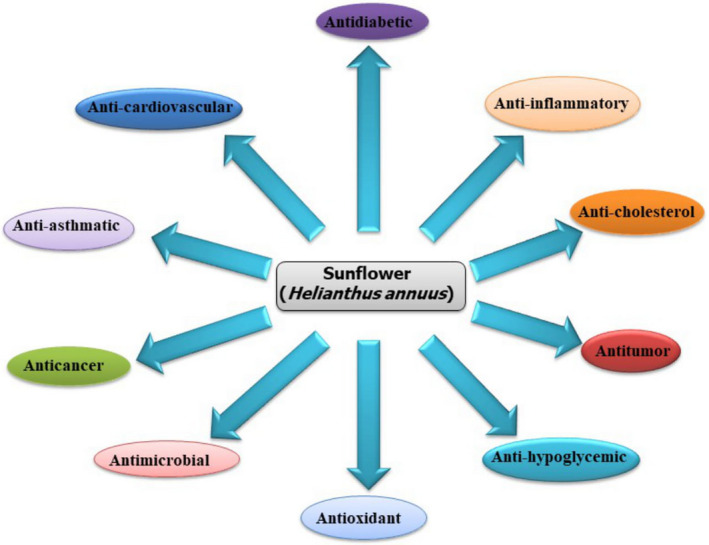
Pharmacological and health benefits of sunflower

The health benefits of sunflower oil, meal, and other products are limitless, as they possess anticancer, antioxidant, antihypertensive, anti‐inflammatory, hypocholesterolemic, skin‐protective, analgesic, and antibacterial activities (Figure 2). Their effects on the muscles, blood vessels, and nerves are also known. Sunflower oil is also efficient in the treatment of colds, coughs, dysentery, constipation, and diseases like urinary, bronchial, pulmonary, and laryngeal infections (Nadeem et al., 2015). Some of the pharmacological and health benefits of sunflower are described below:

### 4.1 Antioxidant benefits

Oxygen is one of the vital elements that involve in various aerobic processes in humans. Antioxidants functions are to protect body cells from damage caused by reactive oxygen species and reduce the risk of developing severe disease conditions such as cataracts, carcinoma, chronic inflammation, atherosclerosis, cardiovascular disease, neurodegenerative diseases, and premature aging (Jiraungkoorskul, 2016). Examples of natural antioxidants from sunflowers, as shown above (Table 3), can help retard or inhibit or prevent oxidation, scavenge free radicals, and thus prevent disease proliferation within the cell (Guo et al., 2017). The use of synthetic antioxidants is limited due to toxicity. Thus, research interest in the discovery of novel antioxidants from plants with currently ongoing might be promising as a source of dietary antioxidants (Singh et al., 2016).

### 4.2 Anti‐inflammatory and cardiovascular benefits

Sunflower is characterized by anti‐inflammatory activity. The seeds of sunflowers are known to be rich in vitamin E and magnesium that serve as an excellent source of fat‐soluble antioxidant to the body (Kumar, Sharma, & Vasudeva, 2017). The antioxidant properties displayed by vitamin E neutralize the free radicals that may cause damage to the fat‐containing structures and molecules, such as cholesterol, brain cells, and cell membranes (Nowicka & Kruk, 2017). The oxidation of cholesterol and attachment of cholesterol particles to the blood vessel walls can cause atherosclerosis, thus resulting in blockage of arteries, stroke, or heart attack. Vitamin E and magnesium exhibit anti‐inflammatory effects, protect biomolecular components, and reduce symptoms like osteoarthritis, rheumatoid, and asthma by scavenging the free radicals tendencies of causing such effects (Bashir, Zahara, Haider, & Tabassum, 2015). The consumption of foods rich in vitamin E and magnesium importantly helps to reduce risk of atherosclerosis, lower blood pressure, rheumatoid arthritis, asthma, osteoarthritis, colon cancer, diabetes, sudden heat sensation in women at menopause, high blood pressure, stroke, heart attack, and cardiovascular disease and migraine headaches (Bashir et al., 2015). The anti‐inflammatory and inhibition effects of chemical compounds triterpene glycosides obtained from the methanol extract of sunflower petals that cause ear edema in induced experimental mice have been reported (Bashir et al., 2015).

Studies have revealed that people who consume more foods rich in vitamin E have a low risk of heart attack than people who inadequately feed on the less vitamin E diet. Poor supply of magnesium can pose high‐risk factors to muscle spasms, high blood pressure, fatigue, soreness, and migraine headaches (Vijayakumar et al., 2016). Therefore, it is recommended that the consumption of sunflower and associated products would greatly reduce the risk of organ diseases and other human infections. Furthermore, sunflower seeds have also proven efficient in the treatment of stomach and esophagus cancers. The research findings on guinea pig according to Berquin et al. (2007) had revealed that the stem marrow and bottom of sunflower flower containing hemicellulose functionally block sarcoma and Ehrlich ascites carcinoma. In some cases, the relationship between cancer development and consumption of oil‐rich food is usually complex and sometimes depends on the characteristics relating to the oil and fat composition in foods.

### 4.3 Anti‐cholesterol benefits

It is believed that certain chemical structure such as phytosterols similar to cholesterol is found in sunflower seeds and the presence of phytosterols in large amount in the human diet tends to reduce blood levels of cholesterol, improve immune response and risk factors to certain disease such as cancers (Farahmandfar, Asnaashari, Pourshayegan, Maghsoudi, & Moniri, 2018). Aside from sunflower seeds, phytosterol is present naturally in oilseed crops such as soybean, pumpkin seeds, rapeseeds, safflower, palm oil, sesame seeds, and pine tree oil (Nagendra Prasad et al., 2012). Their addition to processed foods such as butter, as an alternative food additive, is said to reduce lower cholesterol levels in foods.

Phytosterols, sterols, and tocopherol represent major phytoconstituents in oilseed crops (Sujith‐Kumar, Mawlong, & Singh, 2017). On ingestion, they underwent several mechanisms in lowering the cholesterol levels in the human body, and their appropriate use in clinical, pharmaceutical, and food industries has been recognized to play a major role in human health. Consumption of food with high phytosterols enhances human immunity, proper functioning of the organs, lower cholesterol levels, and even protection against cancer (Moreau et al., 2018). In the real sense, phytosterols can be extracted from plants and added to the processed oils and foods products based on their health and nutritious properties. The standard sunflower oil containing tocopherol and phytosterol is interesting with therapeutic effects on human health as they reduce total plasma cholesterol and low‐density lipoprotein (LDL) cholesterol levels (Rani, Sheoran, & Sharma, 2017).

The major mechanisms by which phytosterol found in sunflower lower the LDL‐cholesterol levels usually occur through the reduction (30%–50%) in the rate of cholesterol absorption in the intestine (Scolaro, de Andrade, & Castro, 2020). The reduction in the cholesterol levels may be achieved by some mechanisms through competition with cholesterol by the solubilization in mixed micelles in the intestinal lumen, thus cause a reduction in the absorbable cholesterol (Blanco‐Vaca, Cedó, & Julve, 2019). Other mechanisms such as reduction of esterified cholesterol in the enterocyte, accelerated removal of cholesterol from the body through the trans‐intestinal cholesterol secretion and modification in the expression of genes encoding proteins that carry sterols have been proposed. Furthermore, the expression of genes encoding proteins has been attributed with Niemann‐Pick C1‐like 1 (NPC1‐L1) protein, reduction in the transport of cholesterol to the enterocyte, or ATP‐binding cassette transporters (ABCG5 and ABCG8), and efflux of cholesterol from the enterocytes to the intestinal lumen (AbuMweis, Marinangeli, Frohlich, & Jones, 2014; Cabral & Klein, 2017).

Consequently, plant sterols function exerts a hypocholesterolemic effect that inhibits the intestinal actions in the absorption of the cholesterol (Jesch & Carr, 2017). The mechanisms of lowering the cholesterol level during the lipid digestion process have been described based on dynamic competition existing between plant sterols and free cholesterol by integrating into the mixed micelles (He et al., 2019). The phytosterols are hydrophobic as compared to cholesterol which is believed to have more affinity for micelles than cholesterol; thus, displacing cholesterol molecules from the mixed micelles and then, the accreted dietary cholesterol is passed through the feces together with recirculating self‐biliary cholesterol, which in turn reduce the absorption and cholesterol level in the living organism (Fumeron, Bard, & Lecerf, 2017). Nevertheless, it has been proposed that the consumption of phytosterol‐containing meals might not be necessary to present simultaneously with cholesterol in the intestinal lumen to inhibit cholesterol absorption. Hence, other anti‐cholesterol mechanisms were suggested.

Other mechanisms of anti‐cholesterol include that the absorption of dietary and free cholesterol into the intestine is normally esterified by an enzyme acyl‐coenzyme A cholesterol acyltransferase 2 (ACAT‐2) prior absorption into the chylomicrons and later extravasate into the lymph (Xu, Du, Turner, Brown, & Yang, 2019). It is evident that phytosterols are poor substances for ACAT‐2 and their interference with esterified cholesterol inside enterocyte could competitively reduce the inhibition potential. Then, the unesterified cholesterol can then be produced to the intestinal lumen by the ABCG5/8 along with free phytosterols with the aid of heterodimer transporter (Ajagbe, Othman, & Myrie, 2015; Cedó, Farràs, Lee‐Rueckert, & Escolà‐Gil, 2019). Therefore, the reduced ACAT activity could then decrease the cholesterol absorption from the intestinal lumen and mixed micelles, once ACAT activity within the enterocyte is regulated by substrate produced. The interaction of phytosterols with intestinal cholesterol sensors (liver X receptor) has been reported to reduce cholesterol absorption in the intestine (Ma et al., 2017).

### 4.4 Anticancer benefits

The health benefits of sunflower are not exclusive to antioxidant, anti‐inflammatory, or cardiovascular effects, and the sunflower is also known to possess anticancer properties. Sunflower seeds are known to be an excellent source of a trace element, selenium. This element is of fundamental importance in improving human immunity against cancerous cells (Roy, Hossan, & Rahmatullah, 2015). The intervention trials of inverse correlation between selenium intake and cancer development using animal models have been reported in many research studies (Cardoso, Duarte, Reis, & Cozzolino, 2017; Khan et al., 2015). The presence of selenium in sunflower has instigated DNA repair and production in degenerated cells, inhibition of growing cancer cells, and induction of apoptosis, self‐destruction of sequence in the body to remove unwanted or worn‐out cells. Furthermore, the incorporation of selenium to the protein active sites such as glutathione peroxidase protects body cells against cancer (Pisoschi & Pop, 2015). Glutathione peroxidase is one of the antioxidant enzymes that support liver function in the detoxification of harmful molecules. At a low concentration of glutathione peroxidase, the toxic molecules found in contact with the cells are not destroyed, hence cause impairment to the cellular DNA and ensuring the growth of cancer cells (Issam, Nawel, & Yassine, 2015). It is believed that due to the selenium richness in sunflower seeds, it can be used as composite foods in the production of good snacks and another domestic diet. Furthermore, the use of sunflower seeds in traditional medicine as an alternative in the treatment of cancer or malignant growth is known (Bashir et al., 2015). However, studies on sunflower seed extract have proven to display anticancer activities against cancer cells.

### 4.5 Antiasthmatic, antidiabetic, and antimicrobial benefits

The use of sunflower in the treatment or reduction in the risk factor of asthma and diabetes has revealed their health benefits. The antiasthmatic and antidiabetic efficacy of sunflower extracts has been reported (Gad & El‐Ahmady, 2018). The oral administration of ethanolic extracts of sunflower seeds extract in rats with antihyperglycemic effect has been reported (Saini & Sharma, 2013). Also, the in vivo antiasthmatic assay of aqueous extract from sunflower on ovalbumin‐induced mice and the assessment of their lungs by hematoxylin and eosin staining had revealed the extract potency in reducing asthma effect on the mice (Kim et al., 2020). Similarly, the consumption of sunflower seed and oil would probably reduce major risk factors of asthma or diabetes in humans. The antimicrobial activity of methanolic sunflower seeds extracts against some pathogenic Gram‐positive and Gram‐negative bacteria, which include *Staphylococcus aureus, S. epidermis, Escherichia coli, Proteus vulgaris,* and *Pseudomonas aeruginosa* that might result in food‐borne illness has been documented (Menzel et al., 2019). It is, therefore, important that sunflower extract can be applied as natural food preservative agents (Thielmann, Kohnen, & Hauser, 2017).

## 5 WORLD PRODUCTION OF SUNFLOWER

The major sunflower‐producing countries in the world include Nigeria, Tanzania, South Africa, Brazil, India, North America, Canada, Argentina, Eastern Europe, China, France, Russia, Spain, and Australia. The Eastern European countries and Argentina produced more than 10% of the world's sunflower seeds. China is the 5th largest sunflower‐producing country in the world, with an annual production of 2.7 million tons. In the years 2013 to 2017, it was reported that the global oilseed crop increased by approximately 15%, with the United States producing approximately 132 million metric tons in 2017, followed by other top oilseeds‐producing countries like Brazil, Argentina, and China (Cheng, Dien, & Singh, 2019). The current world population is expected to rise to 10 billion in 2050, accompanied by a growing demand for foods. To meet this demand, more crop production must increase from 133 million tons to 282 million tons. America and Europe are the major producers of global oilseeds, which account for 60% of world production, while countries like South Africa, Malaysia, and Indonesia produce less quantity of oilseeds. In Europe, France is known to be the first producer of sunflower oil with a production rate of 550.000 tons when compared to other countries like Spain producing 482.000 tons, Hungary producing 390,000 tons, and Romania producing 339.000 tons. Russia, Ukraine, United Emirate, and Argentina account for 82% of global edible oil production (Table 4). Tanzania supplied about 36% National oilseed production of 90,000 tons of edible oil in 2013. The Food and Agriculture Organization of the United Nations (FAO‐UN) has envisaged the prospect of maximum sunflower seed production in Tanzania (Vilvert et al., 2018).

**TABLE 4:** World production of sunflower seeds

Country	Production of sunflower seed (1,000 metric tons)					
	2013/14	2014/15	2015/16	2016/17	2017/18	2018/19
Turkey	1.52	1.48	1.50	1.80	1.80	1.80
Russia	10.20	9.00	10.99	11.60	10.50	12.71
Argentina	2.30	3.00	2.80	3.30	3.40	3.50
Ukraine	10.9	10.3	13.47	13.14	14.60	15.00
South Africa	736	736	755	874	859	740
United State	917	1,005	1,326	1,203	978	961

### 5.1 Sunflower production in South Africa

In South Africa, many farmers engaged in sunflower mechanization farming on vast hectares of land, first by clearing, harrowing, tilling, ridging, application of chemical fertilizers, and pesticides (if any) before planting. Depending on the farm management, some farmers grow only sunflower on farmland while others grow composite crops, that is, maize and sunflower on the same farmland as the case may be.

Mono‐cropping is majorly practiced with a prospect for a bumper harvest. Processing of sunflower seeds by a good number of oil manufacturing industries in South Africa makes sunflower oil readily available in their market. Sunflower meal and husk are domesticated in feeding livestock as a source of energy. Among the cereal grains cultivated in South Africa, sunflower seeds account for 5% of the total production. Planting of sunflower could be attributed based on the price of the commodity substituted for other products such as maize and climatic conditions at a given time. Growing of sunflower is determined by the weather condition in South Africa, simply during winter and summer. More than a decade now, the difference in the average yield (tons/ha) ranges between 0.95 and 1.55 tonnes/ha. Similarly, consumption of processed sunflower seed has progressively increased by 5.9% from December 2012 (572,519 tons) to December 2013 (606,200 tons), hence, making South Africa one of the sunflowers producing country in the world. South Africa is ranked the world's 10th largest producer of sunflower seeds, which accounts for approximately 3.8% of its global production. Sunflowers are major crops grown by farmers in the Southern and Western Free State and the dry parts of the North‐West Province.

It is commonly planted on an open field along roadsides in Mafikeng, North‐West Province, South Africa; an area characterized by low rainfall, dry land, and varied environmental conditions; as irrigation method is used in some commercial farms in growing of sunflower (Rezig, Chouaibi, Meddeb, Msaada, & Hamdi, 2019).

In each of the Nine Provinces in South Africa, namely Limpopo, Boaba (*Adansonia digitata*), Mpumalanga, Barberton Daisy (*Gerbera amesonii*), Kwazulu‐Natal, Crane Flower (*Strelitzia reginae*), Eastern Cape, Cape Aloe (*Aloe ferox*), Western Cape, Grape Vine (*Vitis vinifera*), Northern Cape, Cape Thorn (*Acacia erioloba*), Free State, Orange River Lilly (*Cinumbul bispernum*), North‐West, Sunflower and Gauteng, with no identified plant, have a coat of arms with a plant represented on them. North‐West Province is symbolized with sunflower as the main economic crop grown in the area. This area is characterized as the largest producer of sunflower in South Africa. A few species of sunflowers are grown as ornamentals and usually found in pastures or disturbed areas. The major species known are perennial, with only about a dozen annual species. The crop rotation system is a common phenomenon practiced by farmers growing sunflowers in South Africa, and this helps in the breakage of disease networks, diseases, and insects attack.

Sunflower contributes about 7% gross value of field crops in South Africa; approximately 2% of all agricultural produce in the country. Sunflower cultivation occupies 600,000 ha in South Africa with a 46% cover area in Free State and 39% in North‐West province. It had been documented that out of the total area, 98% of sunflower is produced on dry land, and only 2% is produced by an irrigation system. Sunflower seed production accounts for about 82% of the edible oil produced in South Africa. South Africa remains a net importer of vegetable oil (sunfoil), approximately 219,000 tons' oil extracted from the seeds. The potential socio‐economic importance of sunflowers can be perceived as it can satisfy about 25% of edible oil needed by South Africans and as a possible source of income for the farming population (Torimiro et al., 2014).

Production of sunflower in South Africa on the economic scale is long achieved even if planting conditions are not suitable enough for the growing of other crops let's say soybean, wheat, sorghum, maize, cowpea, etc. Like the sunflower, soybeans account for approximately 3% of the total grains production in South Africa. Its cultivation and production are abundant in KwaZulu‐Natal and Mpumalanga under dryland and irrigation systems. Interestingly, planting of soybean in Eastern and Northern parts of the Free State and the activities of some farmers in the North‐West province has increased the crop yield significantly (Pradhan & Mbohwa, 2014).

## 6 CONSTRAINTS OF SUNFLOWER PRODUCTION

Many challenges facing the agricultural sector are known to have an influenced crop yield and their product, can vary from one country to another due to different weather conditions in season and time. The major constraints affecting sunflower production in South Africa and other countries can be attributed to the shortage of improved seeds varieties to farmers for planting, harsh weather conditions, erratic/low rainfall, unreliable markets, and price fluctuations, high cost of farm input, disease attack, insect‐pest infestation on the plant‐crop, birds attack, lack of farm machinery, unpredictable rainfall, ignorance/lack of awareness, poor extension services, and stiff competition from edible oil imports. As farmers continue planting on farmland in and out of season, this could lead to problems of disease build‐up such as leaf spot and head‐rots, which can easily be controlled by crop rotation, irrigation methods, and the use of biopesticides (Ebrahimian, Seyyedi, Bybordi, & Damalas, 2019).

Changes in climatic conditions have caused a great challenge in the production of sunflower and their yield, as these dictate suitable season for planting certain crops by farmers—favorable weather conditions with adequate rainfall enhanced food production. On the contrary, poor water supply due to erratic rainfall could be problematic for rural farmers that cannot afford the cost of irrigation on their farmland. Although, sunflower cultivation requires low moisture compared to other cereal crops for survival due to its long taproots for the absorption of soil water during the drought. The variation in the climatic conditions from one country to another makes the growth of sunflower more peculiar with the growing season. Irrigation problems can be more perverse in some countries with unsuitable high saline water. Also, water sources from the ground with deep water bed could be more expensive to afford, and this can as well discourage many farmers from engaging in sunflower farming.

On the other hand, high illiteracy, lack of information, or knowledge relating to sunflower cultivation, processing, and marketing can be a challenge to many farmers. Birds, insects, and rodents attack may cause huge loss to the sunflower on the field, and controlling them by physical, chemical, or biological methods is therefore necessary. More also, the fascinating and attractive flower passerby along the sunflower field may pluck the flowers for embellishment purposes, and these can cause huge loss and reduction in the quality and quantity of sunflower output. Furthermore, adequate information about seed rate, the quantity of fertilizers applied, pest control, and marketing is vital for achieving success in sunflower cultivation (Gupta, 2014).

Drought due to water shortage affects crop productivity as sunflower survive more in stress environments than other cereal crops. Sunflower crops are commonly found growing in a limiting water environment; in such a situation, it is expected that making water available through irrigation would drastically increase soil water content with expected increase as situations may warrant. In most cases, the sunflower is often restricted to marginal soil or low water land as compared to maize or soybean that prefer a high moisture environment. Hence, the effects of climate change on sunflower production in most regions of the world could offer new cropping opportunities. In Europe, growing of oilseed sunflowers using chemical fertilizers and pesticides is currently practiced. The less adaptation of some major crops like maize, rice, and wheat in the tropical and temperate regions due to climate change has influenced their yields (Konyalı, 2017).

Diseases and pests attack are critical problems facing sunflower production, not only on the field but also after processing and storage conditions. The transmission of sunflower diseases varies among sunflower cultivars and other crops depending on climatic conditions (dry or wet). The most common diseases affecting sunflower include *Sclerotinia sclerotiorum* (Wilt and Head Rot), rust (*Puccinia helianthi*), root rot (*Sclerotium rolfsii*), leaf spot (*Septoria* and *Alternaria* spp.), charcoal rot (*Rhizoctonia* spp.), white blister (*Albugo tragopogonis*), head rot (*Rhizopus* spp. and *Botrytis* spp.), and stem rot (*Phoma oleracen* var *helianthi‐tuberosi*). Controlling pest infestation on seed crops can, therefore, be reduced by planting hybrid seeds‐resistant cultivars and crop rotation following good agricultural practices (Gontcharov, 2014). More also, planting of sunflower more than three consecutive years on farmlands should be discouraged to reduce the disease build‐up. Furthermore, crop rotation by interchanging sunflowers with other plants such as maize and sorghum would reduce pest infestation. Planting of soybeans, potatoes, groundnut, and common beans with sunflower makes them susceptible to *Sclerotinia* Wilt and Head Rot. The transmission of sunflower disease can be by air (airborne). Farmers are encouraged to use treated seeds for planting, sanitize land to prevent disease build‐up, and the use of disease‐resistant cultivars in disease‐prone areas. It is very unfortunate that till today, no sunflower cultivar has developed resistance to *Sclerotinia* Wilt and Head Rot, and outside this review, further studies can be investigated.

Cumulatively, limiting soil nutrients, low or erratic rainfall, pests attack, an inadequate supply of farm input influenced sunflower productivity. Mono‐cropping system, that is planting of cassava or maize sometimes, causes depletion of soil nutrients. Conversely, the growth of multiple crops interchangeably on farmland would be important in enhancing sunflower production and derivable products. Therefore, major constraints affecting sunflower production can be ameliorated by planting hybrid drought‐resistant sunflower seeds to ensure food security (Gontcharov, 2014).

## 7 FOOD SECURITY AND FOOD VALUE CHAIN

The major key players regarding the sunflower food value chains include the farmers, transporters, marketers (wholesalers and retailers), and consumers. Commercialization of sunflowers as a portion of food by smallholder farmers in selling their farm produce in a local market is usually direct to wholesalers or retailers while large scale farmers sell their products in urban or export markets. Many benefits are linked with the usage of sunflower oil due to their high‐temperature stability, long shelf life, raw materials to industries, and alternative to *trans* fats (Cheng et al., 2019). Most economic crops are potent renewable energy sources. Oilseed crops could be used as an alternative for energy generation, and this could threaten food security in developing countries like South Africa, limiting the amount of food available for human consumption. The use of sunflowers as an alternative for biodiesel could only be profitable when it is produced on a large scale.

The required nutritious food with balanced dietary intake needed for health improvement must be available, accessible, and utilized efficiently. Food insecurity is one of the most potent challenges faced in developing countries. Strategic measures in combating these challenges differ in some countries. In the developing world, among the rural settlement, the prevailing subsistence farming, perhaps due to poor financial strength, lack of essential farm inputs result in food shortage, malnutrition, and hunger (Crush & Frayne, 2011). Thus, the introduction of new intervention agriculture policy into a rural crop value chain would alleviate poverty among rural dwellers. Furthermore, bridging the gap between rural farmers growing sunflowers in improving their livelihood would as well contribute maximally to food security via upgrade in market integration among the sunflower value actors, that is, producers, retailers, wholesalers, and consumers in the rural areas.

## 8 FUTURE PROSPECT/CONCLUSION

Agricultural intensification using microbial resources as a sole commercialized product in sustainable agriculture is promising for the improvement of farm yields and crop productivity. Although, many works of literature have been documented on the use of biofertilizers as alternative use to chemical fertilizers in developing environmental‐friendly agriculture in an ecosystem, positively affect plant growth, yet there is a need for conservation of soil quality in increasing crop biomass. Like legume and cereal plants that required water accessibility for maximum productivity, oilseeds crop such as sunflower can strive well in an environment prone to drought and low rainfall due to their long taproots structure that helps them to withstand harsh environmental conditions. Alternatively, the irrigation system and crop rotation can better enhance sunflower production (Konyalı, 2017). Despite the economic values of most oilseed crops, many constraints, and challenges encompass their production and commercialization. Then, the use of crop rotation in growing sunflowers and other cereal crops has significantly reduced pests or diseases that attack the plants.

Sunflower seed extracts such as sunfoil, meal, and cake could be a promising human diet and livestock feed. The exploitation of sunflower seeds products with high protein content has fond applicable in food processing and various pharmaceutical and agriculture. Therefore, the focus of this review on the use of organic fertilizer in sustainable agriculture could significantly be employed in improving agricultural crop yields and oilseeds production in averting food shortage and high consumption of cholesterol foods that form part of the human diet with diseases‐causing tendencies.

## CONFLICTS OF INTEREST

The authors declare that there is no conflict of interest regarding the publication of this review paper.
